# Role of adiponectin in osteoarthritis

**DOI:** 10.3389/fcell.2022.992764

**Published:** 2022-09-08

**Authors:** Xinyuan Feng, Jiaying Xiao, Lunhao Bai

**Affiliations:** ^1^ Department of Orthopedic Surgery, Shengjing Hospital, China Medical University, Shenyang, China; ^2^ Department of Internal Medicine Integrated Ward 2, Shengjing Hospital, China Medical University, Shenyang, China

**Keywords:** adiponectin, autophagy, apoptosis, pyroptosis, osteoarthritis

## Abstract

Osteoarthritis (OA) is a widespread and most common joint disease which leads to social cost increasing accompany with aging population. Surgery is often the final treatment option. The major progression of OA includes cartilage degradation caused by chondrocytes metabolism imbalance. So, the molecular mechanisms of action in chondrocytes may provide insights into treatment methods for OA. Adiponectin is an adipokine with many biological functions in the cell metabolism. Numerous studies have illustrated that adiponectin has diverse biological effects, such as inhibition of cell apoptosis. It regulates various functions in different organs, including muscle, adipose tissue, brain, and bone, and regulates skeletal homeostasis. However, the relationship between adiponectin and cell death in the progression of OA needs further investigation. We elaborate the structure and function and the effect of adiponectin and state the correlation and intersection between adiponectin, autophagy, inflammation, and OA. From the perspective of oxidative stress, apoptosis, pyroptosis, and autophagy, we discuss the possible association between adiponectin, chondrocyte metabolism, and inflammatory factor efforts in OA. What’s more, we summarize the possible treatment methods, including the use of adiponectin as a drug target, and highlight the potential future mechanistic research. In this review, we summarize the molecular pathways and mechanisms of action of adiponectin in chondrocyte inflammation and death and the pathogenesis of OA. We also review the research on adiponectin as a target for treating OA. These studies provide a novel perspective to explore more effective treatment options considering the complex interrelationship between inflammation and metabolism in OA.

## Introduction

Osteoarthritis (OA) is a degenerative joint disease characterized by pain and disability owing to cartilage damage, synovial inflammation, and joint tissue problems. It is also a significant societal problem, as the disease affects more than 10% of the adult population worldwide (Hunter and Bierma-Zeinstra, 2019). OA is associated with many factors, such as age, sex, trauma, and obesity. One of the most influential and modifiable risk factors is obesity (Wang and He, 2018). Several studies have illustrated a strong association between OA and obesity with an increase in body–mass index (Martel-Pelletier et al., 2016; Reyes et al., 2016; Misra et al., 2019). The mechanisms of how obesity results in the progression of OA are unclear because of the complex interactions among the metabolic, biomechanical, and inflammatory factors that accompany increased adiposity (Collins et al., 2021). Previous studies show that adipokines, which are secreted from fat tissues, are associated with OA (Xie and Chen, 2019).

Adipose tissue has also been deemed as endocrine organ for many years. The adipokines, secreted from adipose tissue, include adiponectin; leptin; resistin; chemerin; adipsin; acylation-stimulating protein (ASP); interleukin (IL)-1β, -6, -8, and -10; and tumor necrosis factor (TNF)-α (Blüher and Mantzoros, 2015; Fasshauer and Blüher, 2015). Adipokines not only regulate appetite, satiety, fat distribution, insulin sensitivity, energy, and inflammation but also modulate adipogenesis and the metabolism and function of adipocytes and immune cells (Blüher, 2014; Olszańska et al., 2021; Rijnsburger et al., 2021). However, the function and potential clinical value of many adipokines remain unknown. Among these different effects, anti-inflammatory effects cannot be overlooked. This review focuses on the effects of adiponectin on OA.

### Adiponectin

Adiponectin is a 30 kDa monomeric glycoprotein, which is secreted in large quantities primarily from the adipose tissue (Berg et al., 2002). The basic structure of adiponectin comprises an N-terminal signal sequence, a nonhomologous or hypervariable region, and a collagenous domain containing 22 collagen repeats (8 Gly-X-Pro and 14 Gly-X-Y), and a C-terminal C1q-like globular domain (Frizzell et al., 2009). It exists in a medium-molecular-weight (hexameric) form and a high-molecular-weight (HMW) form, which are mainly produced *in vivo* (Waki et al., 2003). The formation of hexameric adiponectin is regulated by a disulfide bond between two trimers mediated by the free Cys39. This hexameric form is the basic unit for the HMW form, which comprises 12–18 hexamers arranged in a specific structure (Magkos and Sidossis, 2007). The HMW oligomeric adiponectin is formed by hydroxylation and glycosylation of several highly conserved lysine residues within its collagenous domain. It is also the major bioactive isoform, which leads to insulin sensitization and positively affects cardiovascular health (Pajvani et al., 2004). The disulfide bond formation mediated by Cys-39 in the N-terminal hypervariable region leads to the formation of a multimeric complex, contributing to various biological effects (Pajvani et al., 2003; Waki et al., 2003). Endoplasmic reticulum resident protein 44 (ERp44), a molecular chaperone, located in the endoplasmic reticulum, forms a mixed disulfide bond with adiponectin through the variable region that contains a cysteine residue (Cys36 in humans and Cys39 in mice) (Wang et al., 2007). Adiponectin oligomers are retained in the endoplasmic reticulum (ER) by ERp44 using a thiol-mediated mechanism, but another molecular chaperone, ER oxidoreductase 1-La (Ero1-La), selectively enhances the secretion of HMW adiponectin (Wang et al., 2007).

Mouse and human adiponectin consist of 247 and 244 amino acids, respectively, and have 83% homology (Nakano et al., 1996). Remarkably, the recombinant adiponectin produced by *Escherichia coli* consists of only monomeric adiponectin, which suggests that posttranslational processing by mammalian adipocytes is necessary for the formation of multimeric adiponectin (Wang et al., 2002). The globular domain is similar in structure to that of complement factor C1q, type VIII and X collagen, and TNF-α, which also has biological activity (Hu et al., 1996; Shapiro and Scherer, 1998).

### Adiponectin receptors

Adiponectin receptors are categorized into three types: adiponectin receptors 1 and 2 (AdipoR1 and AdipoR2) and a small adiponectin receptor, T-cadherin (Yamauchi et al., 2003; Hug et al., 2004). AdipoR1 is a high-affinity receptor for globular adiponectin and a low-affinity receptor for full-length adiponectin, which is abundantly expressed in the skeletal muscle, macrophages, and hypothalamus. However, AdipoR2 is an intermediate-affinity receptor for both full-length and globular adiponectin, which is expressed ubiquitously in the liver, white adipose tissue, and vasculature (Yamauchi et al., 2003; Iwabu et al., 2010; Yamauchi and Kadowaki, 2013). T-cadherin shows certain distinct characteristics. The glycosyl inositol (GPI) moiety of T-cadherin keeps it localized to the cell membrane. T-cadherin acts as a receptor for the hexameric and HMW forms of adiponectin (Hug et al., 2004; Parker-Duffen et al., 2013). T-cadherin deficiency causes endothelial dysfunction in type 2 diabetes mellitus (T2DM) vascular segments, indicating that T-cadherin plays a role in T2DM pathogenesis (Wang et al., 2017). Adenosine 5′-monophosphate (AMP)-activated protein kinase (AMPK), Ca2+, PPAR-α, ceramide, and even S1P are found downstream of AdipoR1 and AdipoR2, which serve as major adiponectin receptors and mediate the metabolic activity of adiponectin (Yamauchi et al., 2007; Yamauchi and Kadowaki, 2013).

## Adiponectin and osteoarthritis

Because the occurrence and progression of OA often accompany obesity and other metabolic diseases, it has recently attracted significant attention. It is also significantly associated with synovitis and rheumatoid arthritis. These connections suggest that adiponectin can be used as a novel target for bone tissue metabolic diseases.

### Exercise as anti-inflammatory method

Physical exercise has been verified to exert positive mechanical stress on joints. Appropriate exercise alleviates mild inflammatory conditions in OA, cancer, and other diseases and reduces the complications associated with obesity or a high-fat diet. Studies demonstrate a strong correlation between obesity and some molecules involved in the inflammatory response, such as NF-κB, NLRP3, and caspase-1 (Vandanmagsar et al., 2011; Sun, 2017). Furthermore, NLRP3 inflammasome is differentially affected by different exercise patterns in various pathological factors. Chronic exercise and moderate-intensity and high-intensity interval training inhibit NLRP3 activation, whereas acute exercise activates NLRP3 (Zhang et al., 2021). Another study showed that exercise inhibits NLRP3 inflammasome expression and inhibits inflammation and pyroptosis (Javaid et al., 2021). In addition, swimming attenuated the phosphorylation of NF-κB in aging hippocampus (Lin et al., 2020). And another study found that regular voluntary exercise increase caspase-1 expression to enhanced IL-1β and IL-18 secretion in macrophages (Shirato et al., 2017). Similarly, exercise has been observed to exert different effects on adiponectin. In a resistance training program at various intensities, the elder male participants with low intensities did not observe a change in adiponectin, whereas moderate and high intensity produced an increase in circulating adiponectin levels (Moghadasi et al., 2012). In another study, people who undergo calorie restriction demonstrated an increase total adiponectin concentration while only undertake aerobic exercise did not have this effect (O'Leary et al., 2007). Furthermore, elevated adiponectin promotes IL-6 and IL-8 secretion in Rheumatoid Arthritis (RA) (Choi et al., 2009). That means raising adiponectin levels by exercise exacerbates RA. These findings suggest that different types of exercise have opposite effects on adiponectin metabolism, especially in obesity and other dysfunction diseases such as RA and retinal diseases (Li H. Y. et al., 2019). A study on exercise and diet showed that resistance training in association with healthy food habits can improve some inflammation biomarkers such as insulin-like growth factor 1, adiponectin, leptin, interleukin-6, and interleukin-1β and maintain muscle mass and lessen fat mass in resistance-trained males (Moro et al., 2016). Adiponectin plays an important role in the alleviation of inflammation observed as a result of exercise.

### Adiponectin and oxidative stress in inflammation in OA

The core of oxidative stress is reactive oxygen species (ROS), including free radicals such as oxygen free radicals (OH–), hypochlorite ions (OCl–), superoxide anions (O2–), nitric oxide (NO), and hydrogen peroxide (H2O2). ROS are unstable and highly reactive because of unpaired electrons. They are found at low levels in normal cells and play an essential role in maintaining cellular function and homeostasis (Trachootham et al., 2008). If this physiological mechanism is disrupted, excessive ROS stimulate the gene expression of inflammatory cytokines and chemokines, which causes oxidation of proteins and lipids and changes their functions, ultimately triggering oxidative damage that aggravates the inflammatory response (Lismont et al., 2015). In chondrocytes, low-level ROS often regulate gene expression and the balance between extracellular matrix anabolism and catabolism. Certain cytokines such as IL-1β are also induced by ROS. Furthermore, excessive ROS reduce extracellular matrix synthesis and lead to chondrocyte apoptosis (Ahmad et al., 2020). Therefore, ROS are closely related to cartilage homeostasis.

Adiponectin also plays a crucial role in oxidative stress. It regulates the AMPK/GSK-3β pathway to relieve oxidative stress and inhibits the activation of NLRP3 inflammasome in cerebral ischemia–reperfusion injury (Liu H. et al., 2020). A recent study shows that adiponectin agonist ADP355 activates the Nrf2 and sirtuin 2 downstream pathways, thus reducing myocardial apoptosis and oxidative stress (Zhao et al., 2020). In an acute pyelonephritis mouse model, exogenously administered adiponectin not only elevated adiponectin concentration and lipid content but also had antioxidant effects to reduce arterial stiffness and alleviate renal cell apoptosis and inflammation (Afzal et al., 2021; Dai and He, 2021). Moreover, adiponectin suppressed oxidative/nitrative stress in the arterial endothelium of hyperlipidemic rats (Li et al., 2007). In addition, adiponectin both activates the AMPK signaling pathway and inhibits the NF-kB signaling pathway to resist oxidative stress in cardiomyocytes (Essick et al., 2011). In AdipoR1/AdipoR2 knockdown mice, oxidative stress was elevated (Yamauchi et al., 2007). In human adipose cells, excessive ROS inhibited adiponectin mRNA expression and increased the gene expression of proinflammatory adipocytokines such as IL-6 (Furukawa et al., 2004). Daqian Gu reported that an adiponectin receptor agonist inhibits CIN by limiting oxidative stress and inflammation by activating the downstream AMPK pathway (Furukawa et al., 2004). Because mitochondria produce abundant ROS in cells, when oxidative stress occurs, it decreases adiponectin synthesis in obesity, which is accompanied by mitochondrial dysfunction in adipocytes (Koh et al., 2007). The ADIPOQ gene polymorphism rs1501299 is potentially associated with the risk of developing knee OA (Fernández-Torres et al., 2019). Globular adiponectin induces a proinflammatory response in human astrocytes (Otero et al., 2006). What’s more, adding 0.5 μg/ml adiponectin in ATDC5 mouse chondrocytes, increases in chondrocyte proliferation and the upregulation of type II collagen and aggrecan in chondrocytes, which means adiponectin play a protective role in OA (Challa et al., 2010; Jiang et al., 2022). Thus, these findings indicate that adiponectin is associated with oxidative stress and OA.

### Adiponectin induces apoptosis in OA

Apoptosis (programmed cell death), with the unique characteristic of apoptotic body formation, was first identified by Kerr. Dysregulation of apoptosis is often observed in degenerative diseases such as cancer, obesity, and OA (Kerr et al., 1972). The balance of proteins with opposing apoptotic roles is crucial for the progression of apoptosis, which has been already studied in the context of different diseases (Delbridge and Strasser, 2015).

Both the intrinsic pathway-also named mitochondrial pathway-induced by intracellular signals and the extrinsic pathway-also named the death receptor pathway-triggered by death receptor family proteins and other signals mediate apoptosis (Elmore, 2007). The death receptor proteins, including TNFR, TRAIL receptor 1 and 2, and Fas, contain the death domain (DD), a cytosolic domain, and a cysteine-rich extracellular domain (Ashkenazi and Dixit, 1998). First, Fas and its ligand FasL activate the death-inducing signaling complex, then caspase-8 and caspase-3 are sequentially activated, and eventually, apoptosis occurs (Fuentes-Prior and Salvesen, 2004). Adiponectin is closely associated with apoptosis. In high-glucose–treated human glomerular endothelial cells, AdipoRon, a synthetic adiponectin receptor agonist, reduced oxidative stress induced by high glucose and alleviated endothelial function by activating downstream intracellular Ca2+ signaling (Kim et al., 2018). It is well known that Ca2+ influx may result in mitochondrial dysfunction and activate caspase-3. Therefore, it provides a prospective treatment method for adiponectin and cell apoptosis. Liu et al. found that adiponectin not only activated the AdipoR1/AMPK/PKC pathway to decrease ER stress-induced apoptosis but also inhibited apoptosis by regulating the anti-apoptotic protein Bcl-2 in mouse adipose tissue (Liu Z. et al., 2016). Wu et al. reported that adiponectin induced the restoration of peroxisome proliferator-activated receptor-gamma coactivator-1α-related mitochondrial function and suppressed activating transcription factor 4-CCAAT-enhancer-binding protein homologous protein (CHOP)-induced neural apoptosis (Wu et al., 2020). Thus, adiponectin is potentially involved in cell apoptosis through various pathways.

### Chondrocyte apoptosis

Unlike normal cartilage, osteoarthritic joint cartilage shows an increased rate of chondrocyte apoptosis (Héraud et al., 2000). Mitochondrial activity, microRNA expression, chondrocyte senescence, autophagy, ER stress, and oxidative stress are involved in chondrocyte apoptosis (Engels and Hutvagner, 2006; Ruiz-Romero et al., 2009; Uehara et al., 2014; Mobasheri et al., 2015; Vasheghani et al., 2015), and their mechanisms of action are complex. Osteoarthritic chondrocytes show higher ROS generation, which may promote chondrocyte apoptosis (Ruiz-Romero et al., 2009). Moreover, when the mechanical stress changes, the chondrocyte apoptosis could cause different reactions in animal studies (Loening et al., 2000; Zamli et al., 2013).

Although the intervention of chondrocyte apoptosis is a potentially effective measure to modulate articular cartilage, apoptosis-related drugs, and biological agents may have side effects on the whole system. Pharmacological doses of glucosamine HCl, a nutraceutical for the treatment of OA, were found to induce a decline in the metabolic activity of bovine chondrocytes (de Mattei et al., 2002). IRE1, a key regulator of unfolded protein response in the ER, was reported to have a potential effect on chondrocyte apoptosis. IRE1α deficiency downregulated the prosurvival factors XBP1S and Bcl-2, which increased caspase-3, CHOP, and p-JNK to enhance chondrocyte apoptosis (Huang et al., 2022). This finding provides new insights into the importance of ER stress regulation in OA treatment. Recently, biomaterials have been applied as a practical therapy for OA. Exosomes contain various cytokines and growth factors, which mediate inflammation, enhance cell proliferation, and reduce apoptosis (Lai et al., 2010). Exosomes incorporated into biomaterials for increased targeting and prolonged retention to treat OA enhanced chondrocyte repair and reduced apoptosis effectively (Chen et al., 2022). In a guinea pig OA model, subchondral bone thickening was observed before chondrocyte apoptosis. Regulation of subchondral bone may be a promising treatment strategy in OA (Zamli et al., 2014).

### Pyroptosis and OA

Another form of programmed cell death, pyroptosis, which is caspase dependent and typically accompanied by proinflammatory changes, has been identified in recent years (Fink and Cookson, 2005). The key features of pyroptosis include cell swelling, the release of many proinflammatory factors including IL-1β and IL-18, and inflammasome activation (Liu X. et al., 2016). Danger-associated molecular patterns (DAMPs) or pathogen-associated molecular patterns (PAMPs) are two types of caspase release pattern recognition receptors (PRRs) in pyroptosis (Liu X. et al., 2016). Many studies have demonstrated the role of the NLRP3 inflammasome in osteoarthritis, indicating that NLRP3 is a potential target (An et al., 2020). DAMPs or PAMPs stimulate caspase-1 and macrophages to release NLRP3 and other inflammasomes, which leads to pyroptosis. Proinflammatory cytokines such as IL-1β and IL-18 are accumulated in chondrocytes, and their release is induced by inflammasomes (Man and Kanneganti, 2015). Inflammasomes stimulate chondrocytes to secrete catabolic enzymes, which promote a change in some biomarkers of chondrocytes (Yang et al., 2021). Moreover, NLRP3 also affects the synovial tissue in OA (Zhang et al., 2019a). Pyroptosis may also be associated with the pathological mechanism of pain. As stated before, IL-1β, IL-18, and TNF-α are upregulated in pyroptosis in OA pathology, which increase the sensitivity of joint pain receptors (Mapp and Walsh, 2012), contributing to OA pain.

The relationship between adiponectin and pyroptosis has drawn wide attention in recent years. Ehsan et al. found that adding adiponectin to lipopolysaccharide-stimulated monocytes markedly attenuated lipopolysaccharide-induced expression of NLRP3 inflammasome, cleaved ASC, caspase-1, and IL-1β (pro- and cleaved) (Ehsan et al., 2016), which may be achieved through the modulation of the AMPK, Akt, and NF-κB pathways. Many studies report that adiponectin has an antiatherogenic effects; in coronary atherosclerosis, NLRP3 expression in subcutaneous adipose tissue is negatively correlated with the serum adiponectin level (Bando et al., 2015). Moreover, the adiponectin-AdipoR1 pathway promotes NLRP3 gene expression in renal proximal tubule epithelial cells (Yang et al., 2018). A more recent study shows that adiponectin downregulates NLRP3 *via* miR-711 in Duchenne muscular dystrophy, a skeletal disease. Similarly, APN suppresses the pyroptosis pathway by upregulating miR-133a, which potentially alleviates acute aortic dissection (Duan H. et al., 2020). These findings suggest novel therapeutic approaches for other related disorders (Boursereau et al., 2018). Adiponectin also shows strong effects in cancer. In human breast (MCF-7) and hepatic (HepG2) cancer cells adiponectin exerted potent anti-tumor activity *via* downregulation of estrogen receptor-α expression and blocked leptin-induced estrogen receptor-α activation and suppressed inflammasomes, including NLRP3 and ASC (Raut and Park, 2020). Molecules upstream and downstream of inflammasome pathways, such as ROS, estrogen receptor, and NF-κB, are influenced by adiponectin. Because adiponectin affects different inflammasome pathways, it has the potential to relieve pyroptosis-caused cartilage degradation.

### Adiponectin regulates autophagy in OA

Autophagy refers to the catabolic processes through which the cell turns over its cellular components and damaged organelles. There are three main types of autophagy: 1) macroautophagy (hereafter referred to as autophagy), which involves the formation of a double-membrane vesicle (autophagosome) deputed to sequester damaged organelles and biomolecules, 2) microautophagy, by which the cytosolic material is directly engulfed by the lysosome; and 3) chaperone-mediated autophagy (Kroemer et al., 2010). There are five key stages in autophagy: 1) phagophore formation or nucleation; 2) conjugation of autophagy-related gene proteins ATG5-ATG12, interaction with ATG16L, and multimerization at the phagophore; 3) LC3 processing and insertion into the extending phagophore membrane; 4) capture of random or selective targets for degradation; and 5) fusion of the autophagosome with the lysosome (Glick et al., 2010). The role of autophagy, as a protective mechanism in cells, has been researched in regulating numerous aging-related diseases, including OA. The relationship between aging and OA has been demonstrated in clinical settings and epidemiological research (Rahmati et al., 2017). Recent studies demonstrate that oxidative stress is a crucial factor stimulating autophagy. Mitochondria are the major source of ROS within cells (Brand, 2016). Cellular senescence and apoptosis are strongly correlated with autophagic activity, which may be influenced by oxidative stress (Filomeni et al., 2015).

The main negative regulator of autophagy is the mammalian target of rapamycin (mTOR). It mainly forms two different multiprotein complexes, mTOR complex 1 (mTORC1) and 2 (mTORC2). mTORC1 plays a vital role in the regulation of autophagy (Dikic and Elazar, 2018). TOR kinase is activated downstream of the Akt kinase, PI3 kinase, mitogen-activated protein kinase (MAPK), and AMPK pathways (Sabatini, 2006; Shaw, 2009). Autophagy is inhibited by the AKT and MAPK signaling pathways, whereas the AMPK signaling pathway, as a negative regulatory pathway, promotes autophagy (Sabatini, 2006). Recent studies have focused on systemic or local injection of rapamycin to reduce the symptom of OA *in vivo* (Xue et al., 2017). Hypoxia-inducible factor (HIF)-1α and HIF-2α have shown the potential to treat OA. Because the articular cartilage is maintained in a low oxygen environment, chondrocytes are adapted to hypoxic conditions. Increased HIF-1α and HIF-2α mediate the response of chondrocytes to hypoxia. HIF-1α may protect articular cartilage by promoting the chondrocyte phenotype, maintaining chondrocyte viability, and supporting metabolic adaptation to a hypoxic environment. In contrast with HIF-1α, HIF-2α promotes the expression of catabolic factors in chondrocytes, such as MMP13 and ADAMTS-5 (Zhang et al., 2015). With the microenvironmental changes in chondrocytes, HIF-1 activates AMPK and suppresses mTOR, and chondrocyte autophagy is increased (Bohensky et al., 2010). Two other protein conjugation systems, ATG–microtubule-associated protein light chain 3 (LC3) and the ATG5–ATG12 conjugation system, typically used as autophagy biomarkers, play a role in the elongation of the autophagosome membrane (Ohsumi, 2001). Autophagy is also regulated by the beclin-1 complex, consisting of beclin-1, class III phosphatidylinositol 3-kinase, and ATG14L or UVRAG (Wang et al., 2012). All these factors affect the number and size of autophagosomes in osteoarthritic cartilage.

Adiponectin is also an autophagy-regulating signaling molecule, which exerts its effects by activating AMPK, an upstream marker of autophagy regulation (Kim et al., 2009). AMPK activates uncoordinated 51-like kinase-1 (ULK1), which plays a key role in controlling the autophagic response (Lee and Tournier, 2011). Essick et al. found that adiponectin activates the ERK–mTOR–AMPK signaling pathway to suppress excessive autophagy (Essick et al., 2013). Moreover, adiponectin directly enhances autophagy flux in cardiac myoblasts (Jahng et al., 2015). Adiponectin also mediates the AMPK–mTOR signaling pathway to trigger autophagy (He et al., 2021). In human primary chondrocytes, AdipoRon promotes autophagy to alleviate cartilage calcification in OA (Duan Z. X. et al., 2020). AdipoRon treatment promotes autophagy and improves renal fibrosis in salt-hypertensive mice by activating the AMPK/ULK1 pathway (Li et al., 2021). Notably, in cardiomyocytes pretreated with compound C, the adiponectin treatment did not improve the decreased autophagosome formation but improved the decreased autophagosome clearance induced by β1-adrenergic receptor autoantibody (Sun et al., 2021). Exercise leads to AMPK activation in the muscle in normal mice but not in autophagy-defective mice (Garber, 2012). Exercise also promotes the interaction of Toll-like receptor-9 (TLR9) and beclin-1 to mediate AMPK signaling in skeletal muscle (Liu Y. et al., 2020). Thus, as a crucial downstream molecule of adiponectin, AMPK facilitates the progression of vital pathological diseases, including autophagy in OA.

Adiponectin may promote or inhibit autophagy depending on various factors. One study showed that adiponectin suppressed autophagy by facilitating the expression of p-PI3K, p-AKT, and p-mTOR in a diabetic retinopathy model (Li R. et al., 2019). It was demonstrated that exercise induced the phosphorylation of AMPK and AMPK-dependent ULK1 (Laker et al., 2017). Studies report that the promotion of AMPK activation is affected by exercise duration and intensity (He et al., 2012; Schwalm et al., 2015). Furthermore, the extent of cellular stress, protein damage, and exercise type all influence the autophagic response to exercise (Vainshtein and Hood, 2016). A study showed that in young and old adults, acute resistance exercise reduced autophagic activity in skeletal muscle cells, whereas chronic resistance training increased autophagy regulatory proteins such as ATG5, ATG12, and beclin-1 to enhance autophagy and reduced p62 and the ratio of LC3-II to LC3-I (Fry et al., 2013; Luo et al., 2013). AMPK activation, which is induced by exercise, also inhibits mTOR, thus preventing other diseases such as fatty liver and tumors by promoting autophagy (Guarino et al., 2020).

However, autophagy is a double-edged sword; excessive or uncontrolled autophagy promotes autophagy-dependent cell death (Galluzzi et al., 2018). In malignant tumors, excessive autophagy not only induces a cell death mechanism that leads to the death of drug-resistant tumor cells but also mediates tumor escape and promotes tumor cell survival (Liu W. et al., 2020). Furthermore, excessive autophagy induces cell death in cardiomyocytes, which may cause heart failure (Yu et al., 2015). Appropriate training inhibits excessive autophagy, restores normal autophagy function, and improves cardiovascular disease progression (Chen et al., 2010), but excessive exercise leads to excessive autophagy and causes a negative impact. Studies have reported that high-intensity exercise significantly increases the autophagic activity in cardiomyocytes, causing cardiomyocyte damage and even death (Liu et al., 2017).

### Interplay between autophagy and pyroptosis

The relationship between autophagy and pyroptosis has been verified *via* various methods in many studies. Melatonin induces mitophagy activation to eliminate ROS, thereby repressing NLRP3 inflammasome activation in macrophages (Ma et al., 2018). A similar phenomenon was observed in atherosclerotic plaques; autophagic activity inhibited the activation of NLRP3 and other proinflammatory cytokines to reduce lipid deposition and pyroptosis (Cong et al., 2020). In contrast, repressing autophagy increases NLRP3 inflammasome activation and pyroptosis (Jiang et al., 2018). Moreover, in Taxol-treated nasopharyngeal carcinoma cells, autophagy was activated, and pyroptosis was suppressed, which inhibited the caspase-1/gasdermin D (GSDMD) pathway and inflammasome activation (Wang et al., 2020). In nucleus pulposus cells, autophagy was activated to prevent pyroptosis induced by ROS (Bai et al., 2020). Studies indicate that autophagy inhibits pyroptosis through scavenging of mitochondrial ROS (Sadaf et al., 2020). In addition, rapamycin activates autophagy to reverse GSDMD-mediated pyroptosis and reduces sepsis (Zhuo et al., 2020). Adrenomedullin promotes autophagy through the ROS–AMPK–mTOR signaling pathway, inhibits pyroptosis, and rescues the biological functions of testicular Leydig cells (Li M. Y. et al., 2019).

### Autophagy clear components in pyroptosis

Autophagy leads to degradation of inflammasome components and thus prevents pyroptosis. Curcumin attenuated DOX-induced cardiomyocyte pyroptosis by degrading NLRP3 via a PI3K/Akt/mTOR-dependent pathway (Yu et al., 2020). Toll-like receptors induce PAI-2 and beclin-1 expression by increasing autophagy and NLRP3 degradation to suppress IL-1β maturation (Chuang et al., 2013). Dopamine D1 receptor signaling induces NLRP3 ubiquitination through E3 ubiquitin ligase MARCH7, which leads to autophagy-mediated degradation of NLRP3 (Song et al., 2016). Ubiquitin-specific peptidase 5 (USP5) promotes the autophagic degradation of NLRP3 to attenuate NLRP3 inflammasome activation (Cai et al., 2021). In C57BL/6J mice, galectin-9 facilitates p62-dependent autophagy, degrades NLRP3, and attenuates NLRP3 inflammasome activation in primary peritoneal macrophages (Wang et al., 2021).

### Autophagy may promote pyroptosis

It has been reported that starvation-induced autophagy enhances the extracellular release of IL-1β. However, in bone marrow-derived macrophages, the opposite effect was observed (Dupont et al., 2011). Moreover, in pancreatic beta cells, mono-(2-ethylhexyl) phthalate induced pyroptosis and upregulated autophagy levels, but the increase in autophagy suppressed pyroptosis (Jiang et al., 2021). In ovarian carcinoma cells, osthole scavenged gasdermin E (c-GSDME) and triggered autophagy and pyroptosis, which both induce cell death (Liang et al., 2020).

Adiponectin also mediates pyroptosis in addition to autophagy. In aging mice, NLRP3 inflammasome activity increased insulin sensitivity and the leptin-to-adiponectin ratio and suppressed autophagy flux (Marín-Aguilar et al., 2020). Adiponectin alleviates inflammasome activation and pyroptosis induced by palmitate and decreases ROS production, which are both regulated via the AMPK-JNK/ErK1/2-NFκB/ROS signaling pathway (Dong et al., 2020). Moreover, in human aortic epithelial cells, adiponectin regulated FOXO4, inhibited NLRP3-mediated pyroptosis, and alleviated endothelial dysfunction (Zhang et al., 2019b).

In summary, autophagy mainly negatively affects pyroptosis and alleviates the harmful effects of pyroptosis through key signaling pathways such as AMPK–mTOR and HIF-1α. As mentioned above, autophagy promotes the degradation of inflammasomes, thus attenuating the inflammatory response. Under certain conditions, autophagy induces apoptosis, pyroptosis, and even inflammation. Adiponectin regulation is the key to regulating the effects of autophagy.

## Conclusion

**FIGURE 1:**
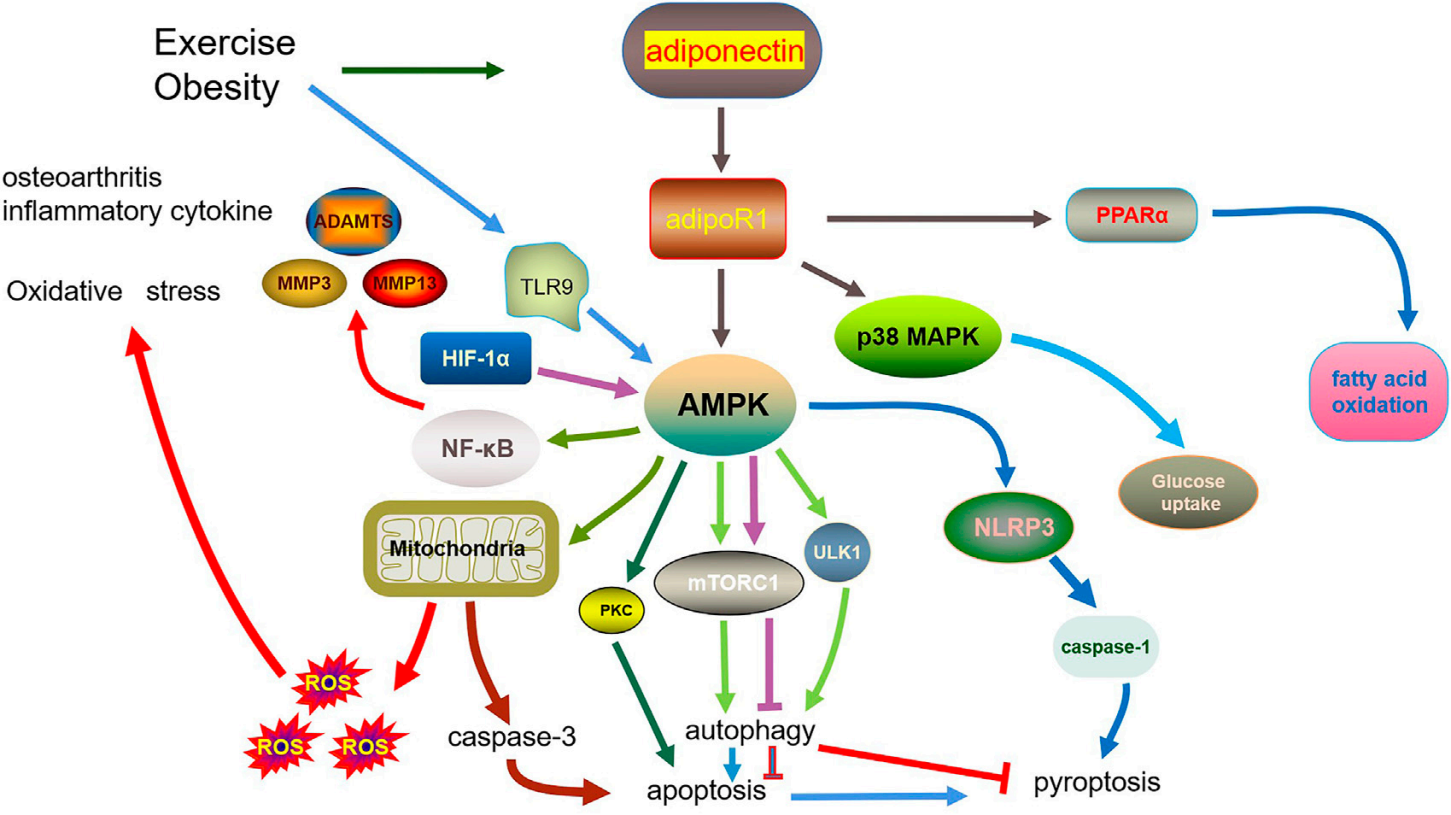
Role of adiponectin in osteoarthritis.

Overall, adiponectin plays an important role in apoptosis, pyroptosis, autophagy, and inflammation in OA. The main characteristic of OA is articular cartilage degradation caused by inflammatory factors. Local and systemic inflammation are associated with the pathogenesis of OA. Proinflammatory cytokines are strongly correlated with adiponectin, which is also involved in OA. This review has summarized the existing research from the perspective of inflammation, oxidative stress, apoptosis, pyroptosis, and autophagy, and their interaction (Figure 1), thus presenting novel strategies for OA treatment and prevention.

Considering that the treatment of OA currently stops at delaying cartilage degeneration, reducing chondrocyte death may be a therapeutic strategy. There are many factors that can affect the death of chondrocytes, such as the release of local inflammatory factors, lipid metabolism. The adiponectin discussed in this article is an important adipokines involved in lipid metabolism. There is a strong relationship between autophagy and oxidative stress and inflammation. However, autophagy is a double-edged sword. Excessive autophagy can promote apoptosis and may also have a negative impact on pyroptosis. Furthermore, the mainstream view considers that intervening at the early stages of OA can protect chondrocytes against a part of cell death.

Focus on the role of adiponectin also can be a treatment strategy. It has a potential to treat metabolism such as OA. To date, numerous studies about using adiponectin and its derivative in this field. Although the effect is limited, the promising is expected. Exercise may be a new way to regulate adiponectin levels in the body. A considerable proportion of patients with osteoarthritis suffer from obesity. Studies have shown that endurance constant-moderate intensity exercise (END) can be a good protection against adiponectin imbalance caused by high-fat diet (Martinez-Huenchullan et al., 2019). In addition, adiponectin derivative CTRPs such as CTRP9 have been found to improve the catabolism and secretion of inflammatory factors in chondrocytes, and effectively reduce the level of IL-18 (Wang et al., 2022). Therefore, regulating the secretion of adiponectin-related metabolic factors may become a future therapeutic direction.

In conclusion, there remains a need for more specific treatment method for OA. Adiponectin is closely related to inflammation and cell metabolism. It can be a promising drug target for OA. However, research into adiponectin and its role in the pathogenesis of OA needs further study. We believe that these thoughts will be realized in future.

## Abbreviations

ASP, Acylation-stimulating protein; AdipoR1 and AdipoR2, Adiponectin receptors 1 and 2; APN, Adiponectin; ATF4, Activating transcription factor 4; Atg, Autophagy-related genes; AMPK, Adenosine 5′-monophosphate (AMP)-activated protein kinase; CHOP, CCAAT-enhancer-binding protein homologous protein; DAMPs, Danger-associated molecular patterns; Ero1-La, ER oxidoreductase 1-La; ER, Endoplasmic reticulum; GPI, Glycosyl inositol; HMW, High-molecular-weight; HIF-1α, Hypoxia-inducible factor 1-alpha; mTOR, Mammalian target of rapamycin; MAPK, Mitogen-activated protein kinase; NO, Nitric oxide; OA, Osteoarthritis; PAMPs, Pathogen-associated molecular patterns; PRRs, Pattern recognition receptors; ROS, Reactive oxygen species; TNF-α, Tumor necrosis factor α; UPR, Unfolded protein response.
